# Morphological and pomological diversity of wild *Prunus microcarpa* Boiss. germplasm

**DOI:** 10.1186/s12870-022-03572-2

**Published:** 2022-04-09

**Authors:** Ali Khadivi, Farhad Mirheidari, Younes Moradi, Simin Paryan

**Affiliations:** Department of Horticultural Sciences, Faculty of Agriculture and Natural Resources, Arak University, 38156-8-8349 Arak, Iran

**Keywords:** Gene pool, Genetic diversity, Conservation, *Prunus microcarpa*, Breeding

## Abstract

**Background:**

*Prunus microcarpa* Boiss. is usually found in dry calcareous and rocky mountain slopes and is well adapted to severe winter and dry-hot summer conditions. Morphological and pomological diversity among 81 accessions of *P. microcarpa* species selected from natural habitats was assessed.

**Results:**

The accessions investigated were significantly different from each other in terms of the traits recorded. Tree growth habit was highly variable, including weeping, spreading, open, semi-erect, and erect. Most of the accessions had very small leaves, a probable adaptation to the xerophytic conditions. Ripening date ranged from mid-June to early August. Fruit weight as the first character considering in domestication process ranged from 0.21 to 0.44 g. Principal component analysis (PCA) could describe the evaluated traits as the 11 main components that were able to justify 76.29% of total variance. Also, the accessions were clustered into two major clusters by the Ward dendrogram.

**Conclusions:**

Significant diversity was revealed, regarding the morphological traits in the evaluated *P. microcarpa* germplasm that reflected the necessity for the conservation of this germplasm, and it is expected that the results gained in this study will assist current *Cerasus* breeding efforts and will maintain the genetic integrity of *P. microcarpa*.

**Supplementary Information:**

The online version contains supplementary material available at 10.1186/s12870-022-03572-2.

## Introduction

Forest trees have hard and long-lasting organisms that thrive in the diverse environments in terms of time and space. Also, such trees are always exposed to various environmental stresses that result from human activities such as pollution, climate change, and habitat fragmentation. To survive these risks and threats, as well as long-term resistance, these plants need to have adaptive potential, which is largely determined by intraspecific genetic diversity [1–3]. Research studies on the conservation of endangered plant species are of great importance to provide management strategies to protect and support biodiversity [3]. Forest genetic resource conservation programs should aim to preserve this diversity [4]. To achieve this goal, awareness of genetic diversity as well as having information about mating and pollen systems and seed dispersal is of great importance. This information provides important insights to establish conservation and restoration programs, such as identifying areas of high diversity, indicating limits for seed collection, and helping breeders to decide on crossbreeding and germplasm management [5] and also helps to design scales of conservation activities [6]. In addition, studies of genetic diversity increase the researcher's awareness of the historical processes that led to the distribution of a plant species, while the conservation of germplasm is highly important to meet future climate change and biotic and abiotic stresses [5].

Genetic diversity in *Prunus* species is highly variable and is related to several factors such as self-fertility or self-sterility, as well as whether it is domesticated or wild. Genetic diversity within a domesticated species is typically less, thus limiting the distribution and production of *Prunus* in specific areas and environmental conditions [7]. Because genetic diversity in the genus *Prunus* is usually low, the use of wild genetic resources is an important way to achieve breeding goals. Also, it is better to use the available gene pool [8]. *Prunus microcarpa* Boiss. is one of the wild stone fruits that is a suitable choice for research. This species is commonly found in rocky and dry mountains at altitudes of 400 to 1800 m and has a very high adaptation with hot summers and cold winters. This species is an important part of the forest along with the resistant species such as oak. Today, most of the natural forests of *P. microcarpa* have been destroyed, and its populations are increasingly declining. Although this plant has a very high resistance to various conditions, but uncontrolled human activities as well as animal stress have caused genetic erosion and this has reduced its diversity [9].

Evaluation of phenotypic structure and diversity is important for determining important traits as well as germplasm collection and level of genetic variation within a species [10, 11]. Morphological traits are clear reactions of genetic diversity. Evaluation of morphological trait is a fast, simple, and inexpensive method that can be used as a general method to estimate the genetic diversity of different plant species morphologically [12, 13]. Evaluation of morphological traits to determine the phenotypic diversity within *Cerasus* subgenus has been performed in some countries and has had significant results [14–17].

Genetic diversity of *Cerasus* germplasm in Iran is under threat due to habitat limitations, diseases, pests, reduced natural regeneration, competition with other species, climate change, pollution, and deforestation. In the present study, phenotypic diversity of wild *P. microcarpa* in the important areas of its distribution in northern and central Iran was studied using morphological traits. The information obtained can be used to establish preservation strategies as well as breeding programs.

## Material and methods

### Plant material

Morphological and pomological diversity among 81 accessions of wild *P. microcarpa* species selected from natural habitats of Isfahan, Mazandaran, and Azerbaijan-e-Gharbi provinces, Iran was assessed. We have permission to collect *P. microcarpa* from Agricultural and Natural Resources, Iran. The plants (either cultivated or wild) including the collection of plant material, are complied with relevant institutional, national, and international guidelines and legislation. The appropriate distances were considered between the accessions in each collection site to avoid the possibility of sampling and collecting clones of the selected accessions. Geographical coordinates and altitude corresponding to surveyed areas are presented in Table 1.

**Table 1:** Geographical description for collection sites of *P. microcarpa* accessions studied

No	Province	Area	Latitude (N)	Longitude (E)	Altitude (m)	Sample size
1	Isfahan	Sokkan	32°58′06″	49°53′44″	2568	18
2	Isfahan	Choghyort	32°57′35″	49°58′12″	2532	12
3	Mazandaran	Kamarbon	36°09′47″	52°19′39″	1577	25
4	Azerbaijan-e-Gharbi	Ghasemloo	37°18′00″	45°07′17″	1449	26

### The characters evaluated

In total, 41 morphological and pomological variables were applied to investigate phenotypic variability among the accessions selected (Table 2). Morphological and pomological evaluations were carried out using 50 replications of leaves and fruits per accession. The traits, including leaf length, leaf width, petiole length, petiole width, fruit length, fruit width, fruit stalk length, fruit stalk diameter, fruit flesh thickness, stone length, stone width, and stone thickness, were measured using a digital caliper. The weight of fruit and stone was measured using an electronic balance with 0.01 g precision. The remaining characters were qualitatively measured based on rating and coding (Table 3) according to the cherry guidelines provided by the International Board for Plant Genetic Resources (IBPGR) [18].

**Table 2:** Statistical descriptive parameters for morphological traits used to study *P. microcarpa* accessions

No	Character	Abbreviation	Unit	Min	Max	Mean	SD	CV (%)
1	Tree growth habit	TGH	Code	1	9	4.88	2.76	56.62
2	Tree growth vigor	TGV	Code	1	5	2.90	1.45	49.86
3	Tree height	THe	Code	1	5	2.98	1.60	53.59
4	Branching	B	Code	1	5	3.05	1.48	48.59
5	Branch density	BD	Code	1	5	3.07	1.39	45.41
6	Branch flexibility	BF	Code	1	5	3.25	1.39	42.83
7	Trunk type	TrTy	Code	1	5	2.93	1.72	58.57
8	Trunk diameter	TrDi	Code	1	5	2.80	1.33	47.39
9	Trunk color	TrC	Code	1	7	2.78	1.95	70.11
10	Canopy density	CaDe	Code	1	5	2.90	1.48	51.03
11	Tendency to form suckers	TeSu	Code	1	5	3.35	1.57	46.99
12	Young shoot spine	YShSp	Code	0	1	0.37	0.49	131.35
13	Young shoot color	YShC	Code	1	5	3.35	1.26	37.52
14	Leaf density	LDe	Code	1	5	3.35	1.67	49.76
15	Leaf length	LLe	mm	7.42	23.08	14.94	4.00	26.76
16	Leaf width	LWi	mm	4.17	14.35	8.67	2.61	30.09
17	Petiole length	PLe	mm	2.09	8.69	4.46	1.46	32.85
18	Petiole width	PWi	mm	0.28	0.87	0.50	0.12	24.44
19	Leaf apex shape	LASha	Code	1	3	2.73	0.69	25.24
20	Leaf color	LC	Code	1	7	3.15	1.33	42.32
21	Leaf shape	LSha	Code	1	3	2.63	0.78	29.73
22	Leaf serration depth	LSeDep	Code	1	5	3.62	1.44	39.70
23	Ripening date	RiDa	Date	Mid-June	Early Aug	4.56	2.98	65.42
24	Fruit density	FrDe	Code	1	5	3.49	1.53	43.95
25	Fruit shape	FrSha	Code	1	5	3.77	1.33	35.15
26	Fruit length	FrLe	mm	7.29	12.62	9.59	1.11	11.61
27	Fruit width	FrWi	mm	5.48	9.06	7.18	0.69	9.58
28	Fruit stalk length	FrStLe	mm	3.38	14.92	7.54	3.28	43.54
29	Fruit stalk diameter	FrStDi	mm	0.32	1.05	0.65	0.14	21.98
30	Fruit weight	FrWe	g	0.21	0.44	0.33	0.05	15.46
31	Fruit color	FrC	Code	1	13	9.35	3.45	36.87
32	Fruit flesh color	FrFlC	Code	1	11	6.73	3.33	49.45
33	Fruit taste	FrTa	Code	1	11	4.06	2.70	66.58
34	Fruit flesh firmness	FrFlFi	Code	1	5	3.17	1.49	47.00
35	Fruit flesh thickness	FrFlTh	mm	1.07	2.35	1.64	0.33	20.37
36	Fruit juice color	FrJC	Code	1	9	5.94	3.12	52.46
37	Stone length	StoLe	mm	5.43	9.62	7.83	0.86	11.00
38	Stone width	StoWi	mm	4.12	6.32	5.10	0.54	10.68
39	Stone thickness	StoTh	mm	3.75	5.32	4.49	0.37	8.33
40	Stone weight	StoWe	g	0.05	0.18	0.12	0.03	24.43
41	Stone shape	StoSha	Code	1	7	3.25	1.43	43.94

**Table 3:** Frequency distribution for the measured qualitative morphological characters in the studied *P. microcarpa* accessions

Character	Frequency (no. of accession)							
	0	1	3	5	7	9	11	13
Tree growth habit	-	Weeping (18)	Spreading (12)	Open (21)	Semi-erect (17)	Erect (13)	-	-
Tree growth vigor	-	Low (23)	Moderate (39)	High (19)	-	-	-	-
Tree height	-	Low (26)	Moderate (30)	High (25)	-	-	-	-
Branching	-	Low (21)	Moderate (37)	High (23)	-	-	-	-
Branch density	-	Low (18)	Moderate (42)	High (21)	-	-	-	-
Branch flexibility	-	Low (15)	Moderate (41)	High (25)	-	-	-	-
Trunk type	-	Multi-trunk/Low (31)	Multi-trunk/Moderate (22)	Multi-trunk/High (31)	-	-	-	-
Trunk diameter	-	Low (22)	Moderate (45)	High (14)	-	-	-	-
Trunk color	-	Dark gray (36)	Gray-black (25)	Dark brown (13)	7	-	-	-
Canopy density	-	Low (24)	Moderate (37)	High (20)	-	-	-	-
Tendency to form suckers	-	Low (19)	Moderate (29)	High (33)	-	-	-	-
Young shoot spine	Absent (51)	Present (30)	-	-	-	-	-	-
Young shoot color	-	Light brown (10)	Brown (47)	Dark brown (24)	-	-	-	-
Leaf density	-	Low (22)	Moderate (23)	High (36)	-	-	-	-
Leaf apex shape	-	Acute (11)	Blate (70)	-	-	-	-	-
Leaf color	-	Light green (10)	Green (60)	Green-silver (6)	Dark green (5)	-	-	-
Leaf shape	-	Ovate (15)	Lanceolate (66)	-	-	-	-	-
Leaf serration depth	-	Low (12)	Moderate (32)	High (37)	-	-	-	-
Ripening date	-	Mid-June (25)	Late June (12)	Early July (13)	Late July (18)	Early August (13)	-	-
Fruit density	-	Low (16)	Moderate (29)	High (36)	-	-	-	-
Fruit shape	-	Round (8)	Cordate (34)	Elongate (39)	-	-	-	-
Fruit color	-	Yellow-orange (2)	Orange (8)	Light red (5)	Red (8)	Red–black (16)	Purple-black (20)	Black (22)
Fruit flesh color	-	Yellow (18)	Dark yellow (1)	Light red (1)	Red (20)	Dark red (36)	Brown (5)	-
Fruit taste	-	Bitter (29)	Very astringent (3)	Astringent (35)	Astringent-sweet (7)	Sweet (5)	Sour–sweet (2)	-
Fruit flesh firmness	-	Low (19)	Moderate (36)	High (26)	-	-	-	-
Fruit juice color	-	Light yellow (19)	Yellow (5)	Dark yellow (1)	Red (31)	Red (25)	-	-
Stone shape	-	Round (11)	Ovate (54)	Oval (11)	Stick (5)	-	-	-

### Statistical analysis

Analysis of variance (ANOVA) was performed to evaluate the variation among accessions based on the traits measured using SAS software [19]. Simple correlations between traits were determined using Pearson correlation coefficients [20]. Principal component analysis (PCA) was used to investigate the relationship between the accessions and determine the main traits effective in accession segregation using SPSS software. Hierarchical cluster analysis (HCA) was performed using Ward’s method and Euclidean coefficient using PAST software [21]. The first and second principal components (PC1/PC2) were used to create a scatter plot with PAST software.

## Results and discussion

The accessions investigated were significantly different from each other in terms of the traits recorded. The CV ranged from 8.33 (in stone thickness) to 131.35% (in young shoot spine). Out of 41 characters measured, the CV in six characters was less than 20.00%, while it was more than 20.00% in 35 characters, and it was more than 50.00% in nine traits (Table 2).

Tree growth habit was highly variable, including weeping (18 accessions), spreading (12), open (21), semi-erect (17), and erect (13). Tree growth vigor, tree height, branching, branch density, branch flexibility, trunk diameter, and canopy density were predominantly moderate. Lanceolate leaf shape was predominant (66 accessions), while leaf apex shape was predominantly blate (70 accessions) (Table 3). The range of leaf-related traits was as follows: leaf length: 7.42–23.08 mm, leaf width: 4.17–14.35 mm, petiole length: 2.09–8.69 mm, and petiole width: 0.28–0.87 mm (Table 2). Most of the accessions had very small leaves, a probable adaptation to the xerophytic conditions, agreed with the previous findings in *P. microcarpa* [22]. Overall, *P. microcarpa* had low leaf area which can indicate better adaptation to drought conditions. This result agreed with previous findings in *Cerasus* [16] and *Amygdalus* [23], who reported that decrease in leaf area is an early adaptive response to water deficit and drought stress [24].

Ripening date ranged from mid-June to early August. Fruit shape was round (8 accessions), cordate (34), and elongate (39). Fruit color showed strong variability, including yellow-orange (2 accessions), orange (8), light red (5), red (8), red–black (16), purple-black (20), and black (22). Also, high diversity was observed in terms of fruit flesh color, ranging from yellow to brown. Fruit taste was highly variable, including bitter (29), very astringent (3), astringent (35), astringent-sweet (7), sweet (5), and sour–sweet (2) (Table 3). The range of fruit-related traits was as follows: fruit length: 7.29–12.62 mm, fruit width: 5.48–9.06 mm, fruit weight: 0.21–0.44 g, and fruit flesh thickness: 1.07–2.35 mm (Table 2). Mohammadi et al. [22] reported that fruit length ranged from 3.70 to 10.47 mm, fruit width varied from 1.94 to 9.64 mm, and fruit weight ranged from 0.17 to 0.90 g in *P. microcarpa*. Fruit weight as the first character considering in domestication process, is very important yield component that can affect the commercial value of fruits for fresh consumption [17]. Fruit stalk length ranged from 3.38 to 14.92 mm, and fruit stalk diameter varied from 0.32 to 1.05 mm. It has been reported that fruit stalk length is one of the most important characteristics for differentiating *Cerasus* germplasm due to its intermediate heritability [15, 17, 25, 26].

**Fig. 1:**
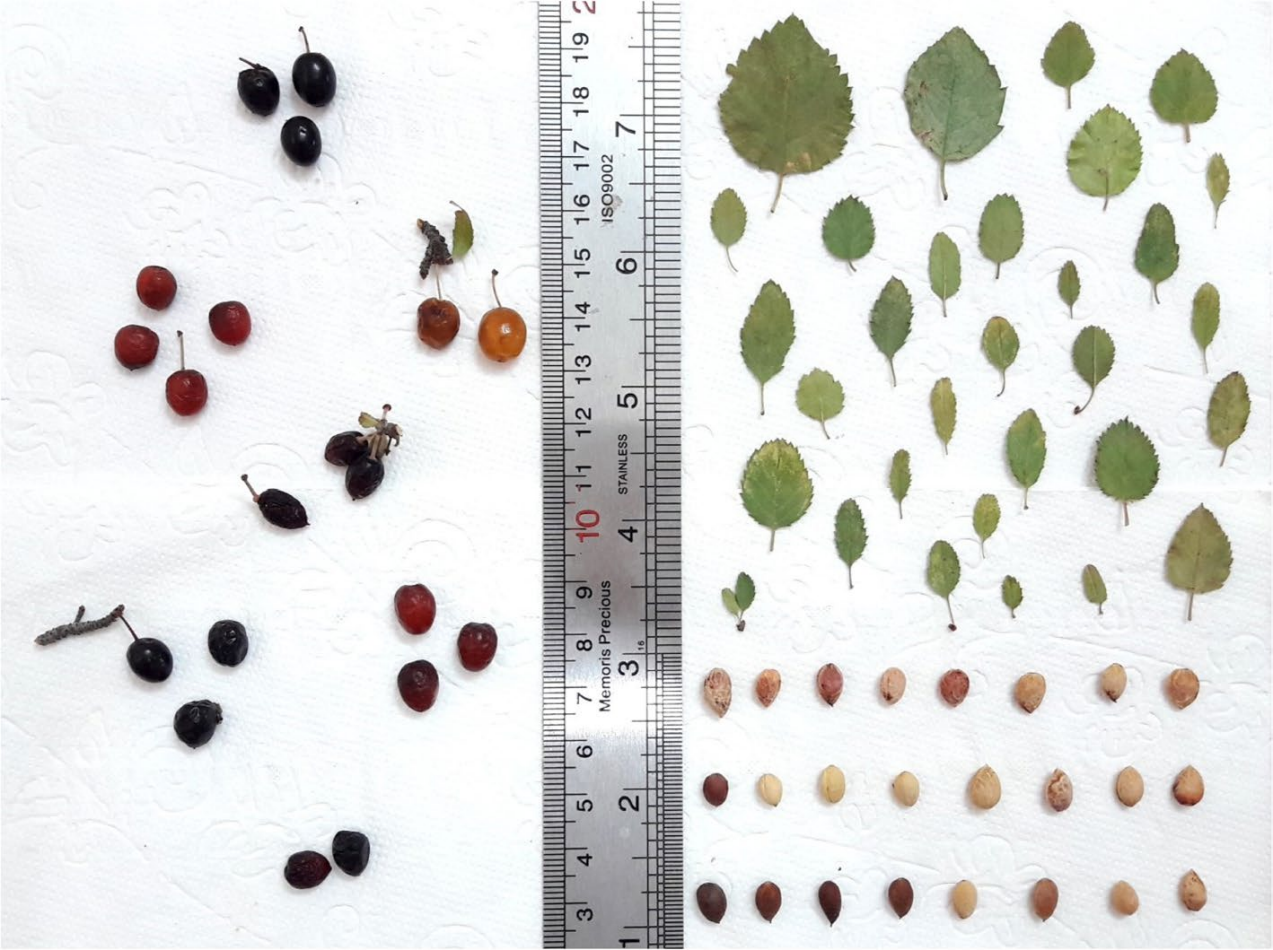
The pictures of leaves, fruit, and stone of *P. microcarpa* accessions studied

Stone shape was round (11), ovate (54), oval (11), and stick (5). The range of fruit stone-related traits was as follows: stone length: 5.43–9.62 mm, stone width: 4.12–6.32 mm, stone thickness: 3.75–5.32 mm, and stone weight: 0.05–0.18 g. Mohammadi et al. [22] reported that stone length ranged from 3.70 to 8.47 mm, stone width varied from 3.70 to 5.99 mm, and stone weight varied from 0.06 to 0.19 g in *P. microcarpa*. The pictures of leaves, fruit, and stone of *P. microcarpa* accessions studied are shown in Fig. 1.

Significant positive or negative correlations were observed between the measured characters (data not shown). Leaf length showed close correlation with leaf width (*r* = 0.69) and agreed with previous work in *Cerasus* [16, 22]. The existence of close positive correlations among leaf traits indicates that more leaf expansion leads to stronger aerial growth. This correlation could be considered as a suitable relationship to improve vigorous rootstocks suitable for dry environments where a fast and strong growth is needed at the beginning of the seasonal life cycle to induce and maintain appropriate vigor in scion and also for reaching to an appropriate size for budding and/or grafting as soon as possible in nurseries [23]. Fruit weight showed positive correlations with fruit length (*r* = 0.65) and fruit width (*r* = 0.66) and agreed with previous work in *Cerasus* [16, 22].

The PCA could describe the evaluated traits as the 11 main components that were able to justify 76.29% of total variance (Table 4). Mohammadi et al. [22] reported the seven main components in the PCA with justifying 72.09% of total variance in *P. microcarpa*. The PC1 was correlated with tree growth habit (0.64), branch flexibility (0.59), trunk color (-0.71), young shoot spine (-0.76), leaf length (0.65), ripening date (-0.68), and fruit taste (-0.77), which explained 12.37% of the contribution of variance. The traits, including branching (0.77), branch density (0.69), canopy density (0.85), the tendency to form suckers (0.71), and leaf density (0.77) were found in the PC2, which accounted for 11.29% of the variance. Five traits, including petiole length (0.73), fruit stalk length (0.53), fruit color (-0.79), fruit flesh color (-0.84), and fruit juice color (-0.84) were placed into the PC3 and included 10.44% of the variance. These components played a major role in distinguishing the accessions studied.

**Table 4:** Eigenvalues of the principal component axes from the PCA of the morphological characters in the studied *P. microcarpa* accessions

Character	Component										
	1	2	3	4	5	6	7	8	9	10	11
Tree growth habit	0.64**	-0.50	-0.19	0.05	0.15	0.30	0.08	0.02	0.01	0.08	-0.13
Tree growth vigor	0.30	-0.14	0.13	-0.01	0.15	0.66**	-0.05	0.24	0.10	0.05	-0.02
Tree height	0.51	-0.27	0.12	0.05	0.09	0.62**	0.11	-0.01	-0.13	-0.04	-0.03
Branching	-0.23	0.77**	-0.09	-0.09	-0.25	0.07	-0.04	0.07	0.00	0.07	-0.11
Branch density	-0.12	0.69**	-0.24	-0.14	-0.31	-0.01	-0.34	0.06	-0.02	0.09	0.05
Branch flexibility	0.59**	0.20	-0.07	0.29	-0.03	-0.25	0.15	-0.05	0.10	-0.10	-0.15
Trunk type	-0.37	0.45	0.31	0.05	0.16	-0.06	0.06	-0.26	-0.22	-0.27	0.23
Trunk diameter	-0.17	0.24	-0.04	0.00	-0.05	0.01	0.05	0.68**	-0.06	0.08	-0.13
Trunk color	-0.71**	0.16	0.01	-0.12	-0.14	-0.12	0.12	-0.01	-0.11	0.17	-0.35
Canopy density	-0.09	0.85**	-0.12	-0.15	-0.11	-0.09	-0.05	0.07	0.01	-0.02	-0.02
Tendency to form suckers	-0.14	0.71**	0.15	-0.04	0.18	0.15	-0.04	-0.09	-0.23	-0.01	0.04
Young shoot spine	-0.76**	0.44	0.04	-0.12	-0.23	-0.29	-0.11	0.04	0.00	-0.03	0.01
Young shoot color	0.06	-0.28	-0.02	0.00	0.04	0.12	0.29	-0.01	0.19	0.19	0.60**
Leaf density	-0.17	0.77**	0.32	-0.20	0.03	-0.08	-0.02	0.09	0.12	-0.13	-0.13
Leaf length	0.65**	-0.25	0.31	0.07	0.13	0.26	0.41	-0.11	-0.03	0.19	0.01
Leaf width	0.51	-0.20	-0.10	0.13	0.00	0.19	0.65**	-0.14	0.04	0.27	0.01
Petiole length	0.37	0.02	0.73**	0.10	0.08	0.24	0.11	0.07	-0.22	-0.20	-0.01
Petiole width	0.09	0.06	0.27	0.57**	-0.32	0.30	0.18	0.11	-0.10	-0.07	0.19
Leaf apex shape	0.11	-0.01	0.10	0.17	-0.08	-0.13	0.02	0.05	0.03	0.81**	0.18
Leaf color	-0.03	0.21	-0.22	-0.25	-0.18	-0.33	-0.18	0.00	-0.08	0.20	0.56**
Leaf shape	0.21	-0.02	0.25	0.16	0.02	0.38	-0.56**	-0.24	0.10	-0.22	0.15
Leaf serration depth	0.19	-0.15	0.08	0.17	0.00	-0.01	0.76**	-0.06	-0.02	-0.18	0.13
Ripening date	-0.68**	0.42	0.42	0.05	-0.12	-0.22	-0.13	0.05	-0.15	-0.16	-0.05
Fruit density	-0.07	0.30	0.08	0.11	0.02	0.67**	0.00	-0.01	0.17	-0.13	0.04
Fruit shape	-0.04	0.08	-0.16	-0.08	-0.10	-0.02	0.08	-0.84**	-0.03	0.05	-0.12
Fruit length	0.06	-0.02	0.40	0.81**	-0.12	-0.12	-0.06	-0.06	-0.16	0.05	-0.04
Fruit width	0.00	-0.28	0.23	0.66**	0.12	0.14	-0.12	0.39	0.00	0.06	0.01
Fruit stalk length	0.14	0.18	0.53**	-0.17	0.52	0.23	0.09	0.02	0.06	-0.35	-0.21
Fruit stalk diameter	0.01	-0.01	-0.17	-0.09	0.14	0.10	0.04	0.04	0.84**	-0.08	0.14
Fruit weight	0.06	-0.19	0.08	0.69**	0.20	0.14	0.20	0.00	-0.01	0.09	0.05
Fruit color	0.24	-0.06	-0.79**	0.10	0.14	0.04	0.01	0.02	0.04	-0.39	0.08
Fruit flesh color	-0.09	0.19	-0.84**	-0.35	-0.08	-0.05	0.04	-0.08	0.03	-0.04	0.04
Fruit taste	-0.77**	0.12	-0.19	-0.07	-0.09	-0.04	-0.06	0.05	0.13	-0.08	-0.02
Fruit flesh firmness	-0.33	0.02	0.09	-0.10	-0.27	-0.46	0.05	0.25	0.02	0.06	0.09
Fruit flesh thickness	-0.28	0.21	0.48	0.41	0.16	0.01	0.01	0.10	-0.48	-0.24	-0.01
Fruit juice color	0.08	-0.10	-0.84**	-0.33	0.07	-0.08	0.05	-0.08	0.14	-0.02	-0.04
Stone length	0.30	-0.17	-0.01	0.74**	0.06	-0.03	0.04	-0.10	0.13	0.07	-0.22
Stone width	0.05	-0.22	0.03	0.39	0.78**	0.13	-0.06	-0.04	-0.10	0.06	0.01
Stone thickness	0.27	-0.11	-0.06	-0.10	0.79**	0.14	0.02	0.08	0.07	-0.14	0.00
Stone weight	0.40	-0.16	-0.07	0.53**	0.49	0.09	0.11	0.07	0.08	0.06	-0.02
Stone shape	-0.11	-0.11	-0.13	0.29	-0.38	0.13	-0.22	-0.13	0.59**	0.23	-0.18
Total	5.07	4.63	4.28	3.82	2.66	2.56	2.01	1.72	1.71	1.61	1.22
% of Variance	12.37	11.29	10.44	9.32	6.48	6.25	4.90	4.19	4.17	3.92	2.97
Cumulative %	12.37	23.66	34.10	43.41	49.89	56.14	61.03	65.22	69.39	73.32	76.29

^**^ Eigenvalues ≥ 0.53 are significant at the *P* ≤ 0.01 level

**Fig. 2:**
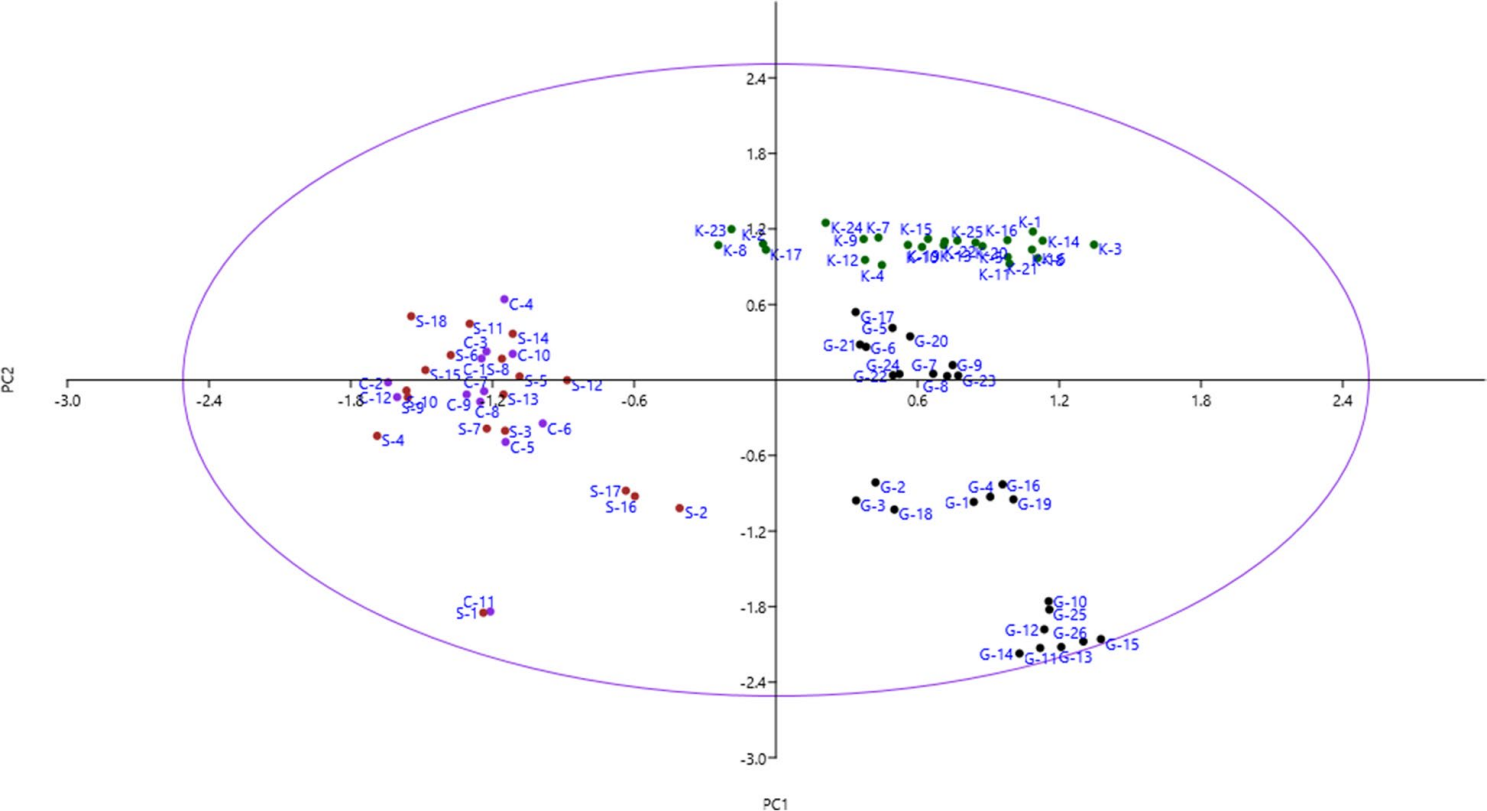
Scatter plot for the studied *P. microcarpa* accessions based on PC1/PC2. The symbols represent the accessions of each area in the plot, including Choghyort (C), Sokkan (S), Kamarbon (K), and Ghasemloo (G).

**Fig. 3:**
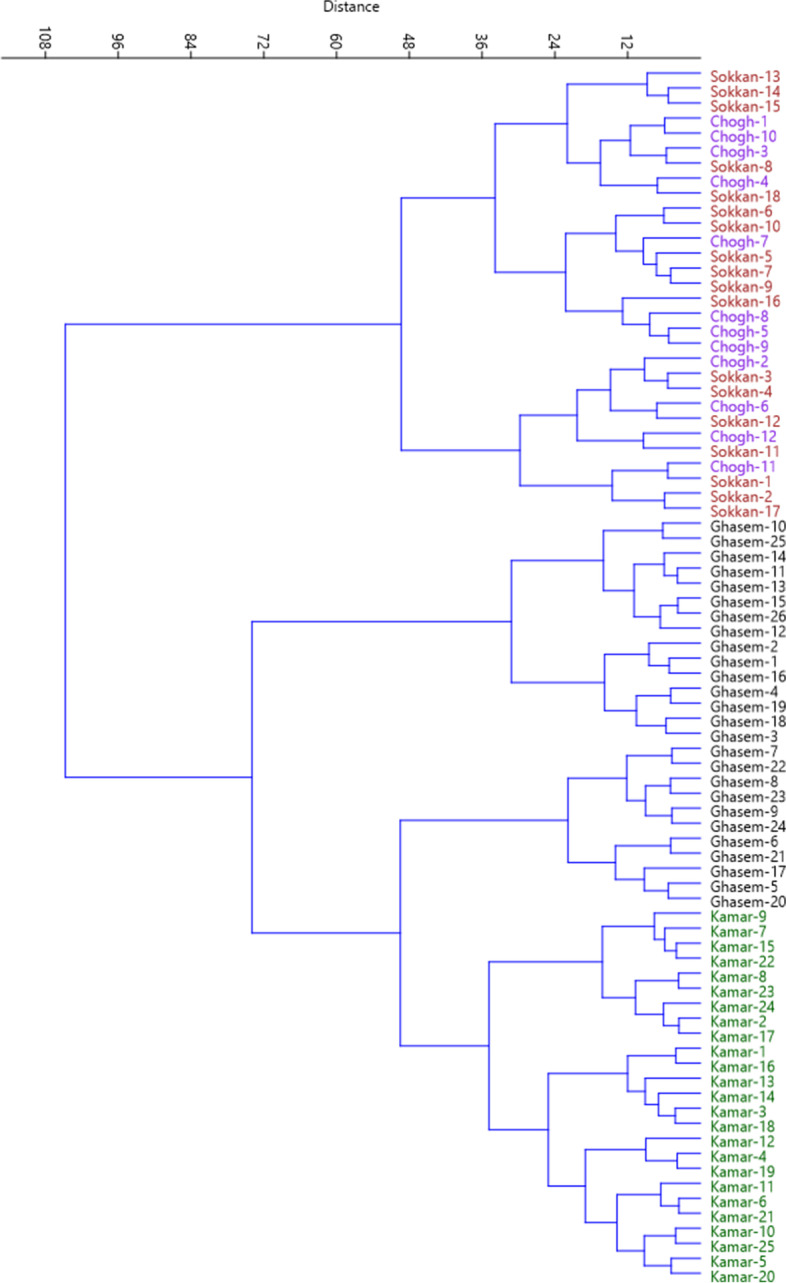
Ward cluster analysis of the studied *P. microcarpa* accessions based on morphological traits using Euclidean distances

**Fig. 4:**
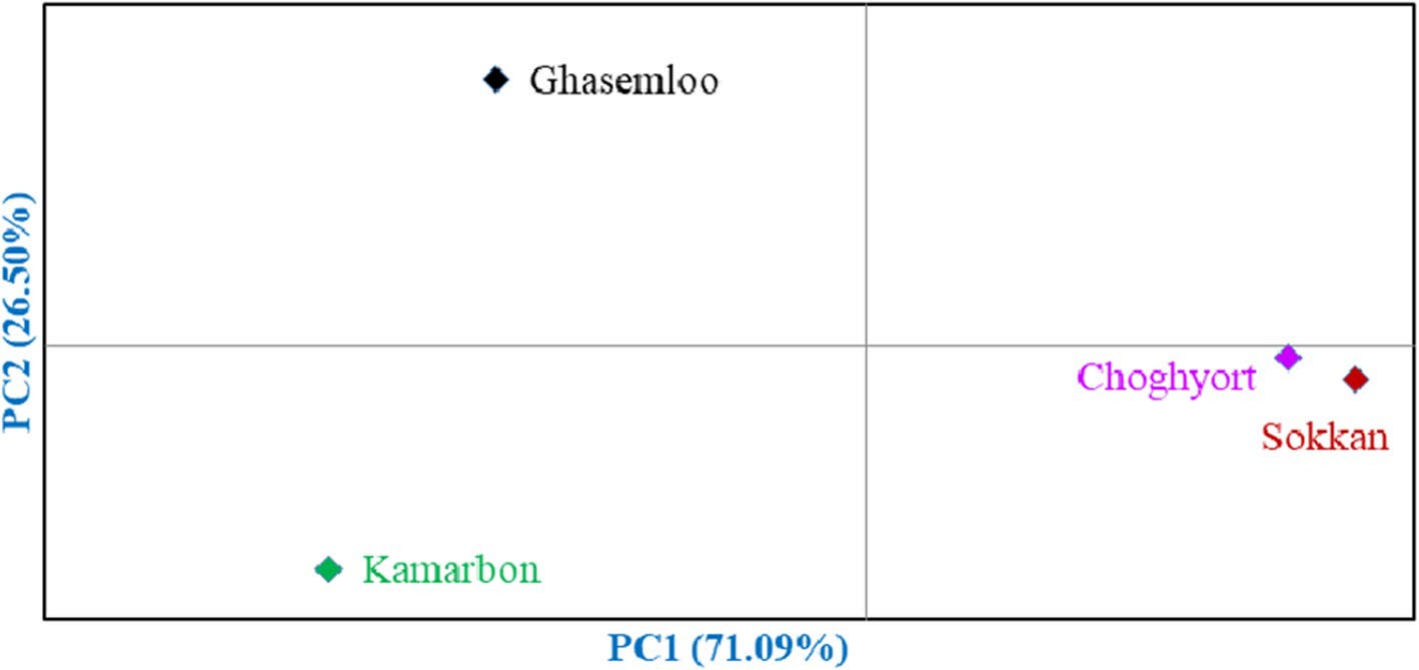
Bi-plot for the studied populations of *P. microcarpa* based on the morphological characters

Bi-plot analysis was performed using PC1 and PC2 which accounted for 23.66% of the variance (Fig. 2). The accessions that were in close proximity were more similar in terms of effective traits in PC1 and PC2 and were placed into the same group. Also, the accessions were clustered into two major clusters by the Ward dendrogram (Fig. 3). The first cluster (I) was divided into two sub-clusters. Sub-cluster I-A included 19 accessions, while 11 accessions were grouped into sub-cluster I-B. The remaining accessions were placed into the second cluster (II), forming two sub-clusters. Sub-cluster II-A included 15 accessions, while 36 accessions were grouped into sub-cluster II-B. Besides, population analysis showed that the studied areas were divided into three main groups (Fig. 4). Group I included Ghasemloo area, while group II included Kamarbon area. Group III consisted of Choghyort and Sokkan areas so that the geographical distance is low among these two populations and gene flow may occur among them [26].

Morphological characterization is the first step in plant resource discovery and conservation [27]. The evaluation of phenotypic variation is also crucial in determining adaptation, agronomic potential and breeding value of landraces [28]. Many successful studies have suggested that high diversity in morphological traits could be a useful tool for the *Prunus* germplasm [14, 16, 22, 23, 26].

The main objective in any plant genetic resource conservation program should be to maintain the highest possible level of genetic variability [29]. The richness of genetic and phenotypic variation in the wild species makes them the most important reservoirs of breeding resources. Unfortunately, anthropogenic activities have significantly influenced the natural habitats and given rise to the dramatically disappearance of the germplasm [30]. These activities will inevitably affect population regeneration and also hinder resource conservation and economic development. Thus, it is recommended to combine measures of conservation (ex situ and in situ) to preserve these valuable genetic resources. Firstly, the construction of a core germplasm repository is absolutely essential. This will allow the ex situ conservation of certain rare individuals and permit the collection of germplam resources in greater breadth [26].

## Conclusions

The results of the study contribute to a better understanding of genetic variation of wild *P. microcarpa* germplasm in Iran, including efforts for preserving biodiversity. Furthermore, the present findings give useful indications on how to act for more rational planning of the management of reproductive material. Significant diversity was revealed, regarding the morphological traits in the evaluated *P. microcarpa* germplasm. This diversity allows the effective parental selection in various breeding programs, referring to fruit quality and aiming at different aspects of postharvest utilization, besides high yield and resistance to diseases. The high genetic diversity observed within *P. microcarpa* species reflected the necessity for the conservation of this germplasm, and it is expected that the results gained in this study will assist current *Cerasus* breeding efforts and will maintain the genetic integrity of *P. microcarpa*.

## Supplementary Information

**Additional file 1.** 

## Data Availability

It was provided as supplementary file.
